# A new understanding of *Acanthamoeba castellanii*: dispelling the role of bacterial pore-forming toxins in cyst formation and amoebicidal actions

**DOI:** 10.1038/s41420-025-02345-8

**Published:** 2025-02-19

**Authors:** Abdelbasset Yabrag, Naeem Ullah, Palwasha Baryalai, Irfan Ahmad, Nikola Zlatkov, Eric Toh, Toril Lindbäck, Bernt Eric Uhlin, Sun Nyunt Wai, Aftab Nadeem

**Affiliations:** 1 Department of Molecular Biology and Umeå Centre for Microbial Research (UCMR), Umeå University, SE-90187 Umeå, Sweden; 2 The Laboratory for Molecular Infection Medicine Sweden (MIMS), Umeå University, SE-90187 Umeå, Sweden; 3 Department of Pathology, Medical University of Vienna, Vienna, Austria; 4 Department of Paraclinical Sciences, Faculty of Veterinary Medicine, Norwegian University of Life Sciences, Ås, Norway; 5 Present Address: Department of Cell and Molecular Biology, Science for Life Laboratory, Uppsala University, SE-75123 Uppsala, Sweden

**Keywords:** Environmental microbiology, Bacterial infection

## Abstract

Pore-forming toxins (PFTs) are recognized as major virulence factors produced by both Gram-positive and Gram-negative bacteria. While the effects of PFTs have been extensively investigated using mammalian cells as a model system, their interactions with the environmental host, *Acanthamoeba castellanii* remains less understood. This study employed high-throughput image screening (HTI), advanced microscopy, western blot analysis, and cytotoxicity assays to evaluate the impact of PFT-producing bacterial species on their virulence against *A. castellanii*. Our unbiased HTI data analysis reveals that the cyst induction of *A. castellanii* in response to various bacterial species does not correlate with the presence of PFT-producing bacteria. Moreover, *A. castellanii* demonstrates resistance to PFT-mediated cytotoxicity, in contrast to mammalian macrophages. Notably, *Vibrio anguillarum* and *Ralstonia eutropha* triggered a high frequency of cyst formation and cytotoxicity in infected *A. castellanii*. In summary, our findings reveal that *A. castellanii* exhibits a unique resistance to PFTs, unlike mammalian cells, suggesting its potential ecological role as a reservoir for diverse pathogenic species and its influence on their persistence and proliferation in the environment.

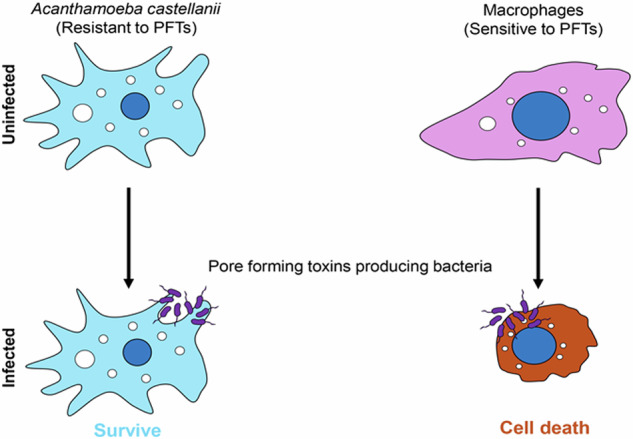

## Introduction

Pore-forming toxins (PFTs) are a diverse group of proteins found across different kingdoms of life, playing a crucial role in the study of cellular processes [1]. In pathogenic bacteria, these toxins are well-established virulence factors, produced by both Gram-positive and Gram-negative pathogenic bacteria during host infections [2]. The release of PFTs by these bacteria is pivotal in facilitating either the spread or colonization within the host [3]. Structurally, PFTs are classified into two main types: α-pore-forming toxins (α-PFTs) and β-pore-forming toxins (β-PFTs). These toxins are secreted as soluble forms, and upon interaction with the target cell lipid membrane, they may undergo conformational changes. The insertion of PFTs into the target cell membrane triggers their oligomerization resulting in the formation of pores or channels that are crucial for mammalian cell lysis [1]. The α-PFTs include a single component toxin such as ClyA from *Escherichia coli* [4], two component toxins like YaxAB from *Yersinia enterocolitica* and three component toxins such as Hbl-BL_1_L_2_, NheABC from *Bacillus cereus* [5], MakABE from *Vibrio cholerae* [6], AhlABC from *Aeromonas hydrophila* [7], and SmhABC from *Serratia marcescens* [8]. Two examples of well characterized single-component β-PFTs are the *Vibrio cholerae* cytolysin (VCC) [9], and listeriolysin O (LLO) produced by *Listeria monocytogenes* [10].

The grazing behavior of amoebae on microorganisms is a fundamental aspect of both aquatic and soil ecosystems [11]. *Acanthamoeba* species exhibit a dynamic life cycle, alternating between an active, vegetative trophozoite stage and a dormant cyst stage, depending on environmental conditions [12]. *Acanthamoeba castellanii* has been extensively studied for its interaction with both human host and various bacterial species [13]. Pathogenic *Acanthamoeba* species are known as causative agents of various human diseases, including keratitis, granulomatous amoebic encephalitis (GAE), sinusitis and disseminated cutaneous diseases [14]. In addition, *A. castellanii* plays a significant ecological role by preying on various bacterial pathogens including *Mycobacteria* spp, *E. coli*, *V. cholerae*, *Vibrio parahaemolyticus*, *Campylobacter jejuni*, *Pseudomonas aeruginosa*, *Salmonella typhimurium, Legionella pneumophila*, *B. cereus* and *Francisella tularensis* [15–19]. Despite its predatory nature, certain bacterial species can resist *A. castellanii* predation and survive intracellularly without harming the amoeba host [15, 17, 18]. This predator-prey interaction presumably drives the development of adaptive traits in bacteria, often enhancing their survival and adaptability in various ecological niches [19, 20]. Furthermore, it is plausible that some bacterial species and *A. castellanii* have coevolved due to frequent encounters in various environments [15, 17, 18]. Upon encountering *A. castellanii*, pathogenic bacterial species may gain or attenuate their virulence for the target host [20]. Some bacterial pathogens may utilize PFTs as virulence factors to counteract environmental predators like *A. castellanii* or to lyse mammalian host cells [2, 6, 21–23]. Such interactions highlight the amoeba’s possible role as a transient host, potentially aiding the survival and spread of various bacterial pathogens in diverse environmental conditions.

Quantitative cell biology requires the measurement of diverse cellular properties, including cellular morphology, RNA, and protein expression. To obtain this information at the individual cell level, image segmentation is essential, often relying on staining for cellular markers [24, 25]. Numerous cell segmentation tools are available for analyzing high-resolution microscopy images over small fields of view. These tools offer a balance between flexibility and automation, but their application becomes challenging when dealing with large datasets typical of high-throughput image (HTI) screening, which require segmenting individual cell features from the entire field of view under specific experimental conditions [24–26]. Some segmentation methods involve manual labeling that is time-consuming and impractical for the screening of large datasets, while other methods are fully automated based on deep neural networks with parameters estimated on large training datasets [24, 25, 27, 28]. While automated methods offer advantages like reduced manual effort, enhanced reproducibility, and scalability, they often require specialized training datasets and face difficulties in generalizing across different image types. Consequently, these methods typically require new human-labeled datasets to optimize performance for specific image types. Machine learning algorithms, like Cellpose and StarDist, provide promising solutions for analyzing HTI data, enabling batch processing that saves time and improves reproducibility [25, 28].

The present study evaluates the impact of PFTs produced by various bacterial species on the viability and cyst induction of *A. castellanii*, employing HTI screening techniques. Our findings highlight a notable difference in the response of mammalian cells and *A. castellanii* to PFT-producing bacterial species. While most PFT-producing bacterial species exhibited lethal effects on mammalian cells, *A. castellanii* retained its viability under comparable experimental conditions, highlighting its distinct resistance to bacterial PFTs. Moreover, we investigated the correlation between the PFT-producing bacteria and cyst formation in *A. castellanii*. Our findings suggest no direct correlation, indicating that the presence of these bacteria does not necessarily trigger cyst formation in the infected amoebae. To further investigate these interactions, we examined the cytotoxicity of *B. cereus*, known for its potent activity against mammalian macrophages, and compared its effect with *B. cereus* isogenic mutant Δ*nheBC* lacking the NheB and NheC components in its tripartite pore complex. While the mutant Δ*nheBC* strain exhibited reduced cytotoxicity against mammalian cells, both the wild type and the Δ*nheBC* mutant strain failed to induce cytotoxicity in *A. castellanii* under similar conditions. Our findings reveal that *A. castellanii* has acquired resistance mechanisms against a broad range of PFT-producing bacterial species, thereby distinguishing it from mammalian cells in terms of susceptibility.

## Results

### Absence of correlation between bacterial pore-forming toxins and cyst induction in *Acanthamoeba castellanii*

**Fig. 1:**
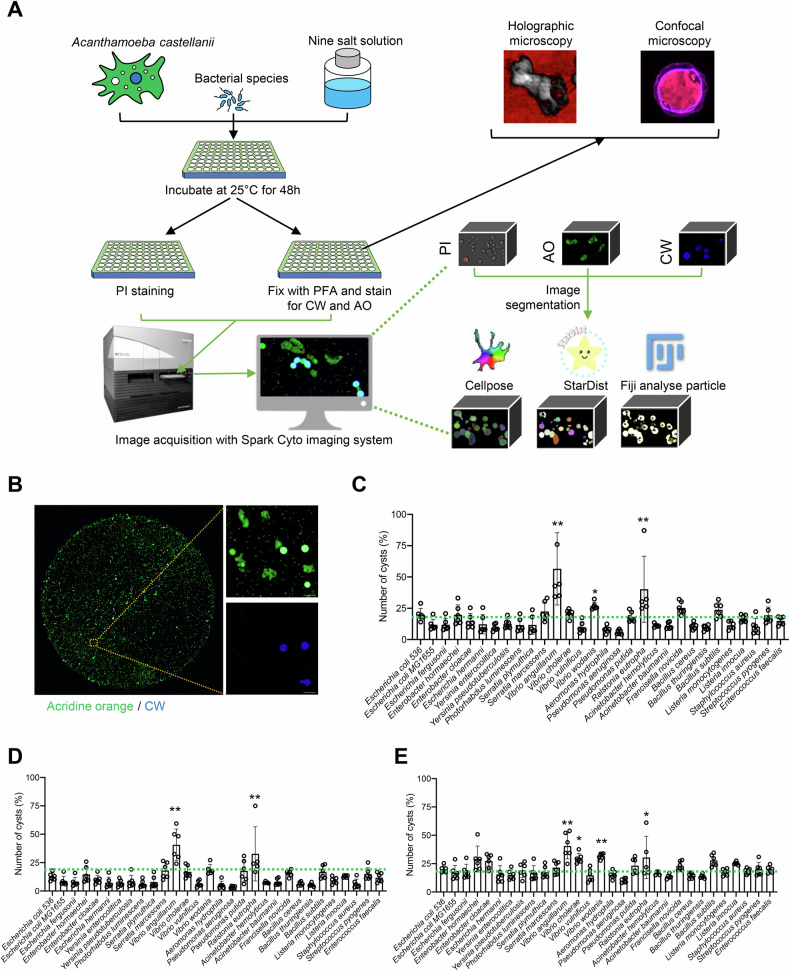
Cysts induction of *A. castellanii* is not correlated with bacteria that produce PFTs. **A** Schematic overview of *A. castellanii* infected with various bacterial species in a 96-well plate. **B** *A. castellanii* were infected with various bacterial species at an MOI of 200 in NSS media for 48 h, followed by fixation and staining with acridine orange (AO, green) and calcofluor white (CW, blue). Images were acquired using a Spark Cyto imaging system for the entire well of the 96-well plate, followed by image segmentation using (**C**) Cellpose, (**D**) StarDist, or (**E**) Fiji analyze particles. The total number of CW-positive cysts was normalized against the total number of *A. castellanii* stained with AO. The histogram indicates the number of *A. castellanii* cysts induced by various bacterial species. Each data point represents five to six replicates from two biologically independent experiments; the bar graphs show the mean ± s.d. Significance was determined from replicates using a one-way analysis of variance (ANOVA) with Dunnett’s post-test against *E. coli* MG1655. **p < 0.01, *p ≤ 0.05.

To investigate the potential contribution of PFTs to the formation of cysts in *A. castellanii*, we developed a high-throughput imaging (HTI) screening method. This method enables objective data analysis of *A. castellanii* cultures infected with various bacterial species (Fig. 1 and Supplementary Fig. 1). The samples were stained using acridine orange, which labels both cysts and trophozoites, and calcofluor white (CW), which binds to cellulose and specifically stains cysts [22]. To ensure unbiased data analysis, we acquired images of the entire 96-well plate using the Spark Cyto imaging system and analyzed the images using three open-source image analysis tools. These included the Fiji plugin “Analyze Particles” and two advanced deep-learning-based segmentation tools, namely, Cellpose 2 and StarDist [25, 27, 28]. These tools were utilized to evaluate their efficiency in segmenting both trophozoite and cysts in *A. castellanii* (Fig. 1C–E and Supplementary Fig. 1A).

In comparing segmentation algorithms, Cellpose 2 and StarDist demonstrated markedly enhanced efficiency in delineating cell clusters compared to the “Analyze Particles” method, which produced suboptimal results. Specifically, the Fiji plugin “Analyze Particles” failed to effectively segment trophozoites present in clusters (Supplementary Fig. 1A). In contrast, Cellpose 2 accurately identified *A. castellanii* trophozoites and cysts as solitary cells. The StarDist algorithm, primarily designed for segmenting round objects, identified cysts as individual objects but has difficulty in segmenting trophozoites, which vary in shape within the *A. castellanii* population. When using StarDist, a single trophozoite was often incorrectly segmented into multiple objects (Supplementary Fig. 1A). Among these three algorithms, Cellpose 2 consistently provided the most accurate and reliable segmentation results (Supplementary Fig. 1A). Among the tested bacterial species, *Vibrio anguillarum* (aquatic bacterium) and *Ralstonia eutropha* (found in both soil and aquatic environments) induced the highest number of cysts, as detected by all three algorithms used in this study. In contrast, environmental bacteria known for producing PFTs, such as *Serratia marcescens*, *V. cholerae*, *Pseudomonas aeruginosa*, *Bacillus cereus*, *Bacillus thuringiensis*, and *Listeria monocytogenes*, induced relatively few cysts in *A. castellanii* (Fig. 1C–E). Notably, the Fiji “Analyze Particles” tool identified a significant increase in number of cysts produced by *V. cholerae* and *V. wodanis*, almost reaching levels similar to those induced by *R. eutropha*. However, the deep-learning algorithms Cellpose 2 and StarDist consistently identified *V. anguillarum* and *R. eutropha* as the primary cyst-inducing bacteria.

Within the same bacterial family, different species exhibited differential impacts on cyst induction in *A. castellanii*. For example, while *V. cholerae* had a lesser effect on cyst formation, *V. wodanis* showed a more pronounced induction. Similarly, among *Bacillus* species, *B. cereus* and *B. thuringiensis* were less effective in inducing cysts compared to *B. subtilis*. In our HTI screen, we also included the urinary tract infection isolate *E. coli* 536, known for its rapid lysis of mammalian cells via PFTs [29]. However, this strain did not significantly induce cyst formation in *A. castellanii*, when compared to non-pathogenic *E. coli* MG1655 (Fig. 1C–E).

**Fig. 2:**
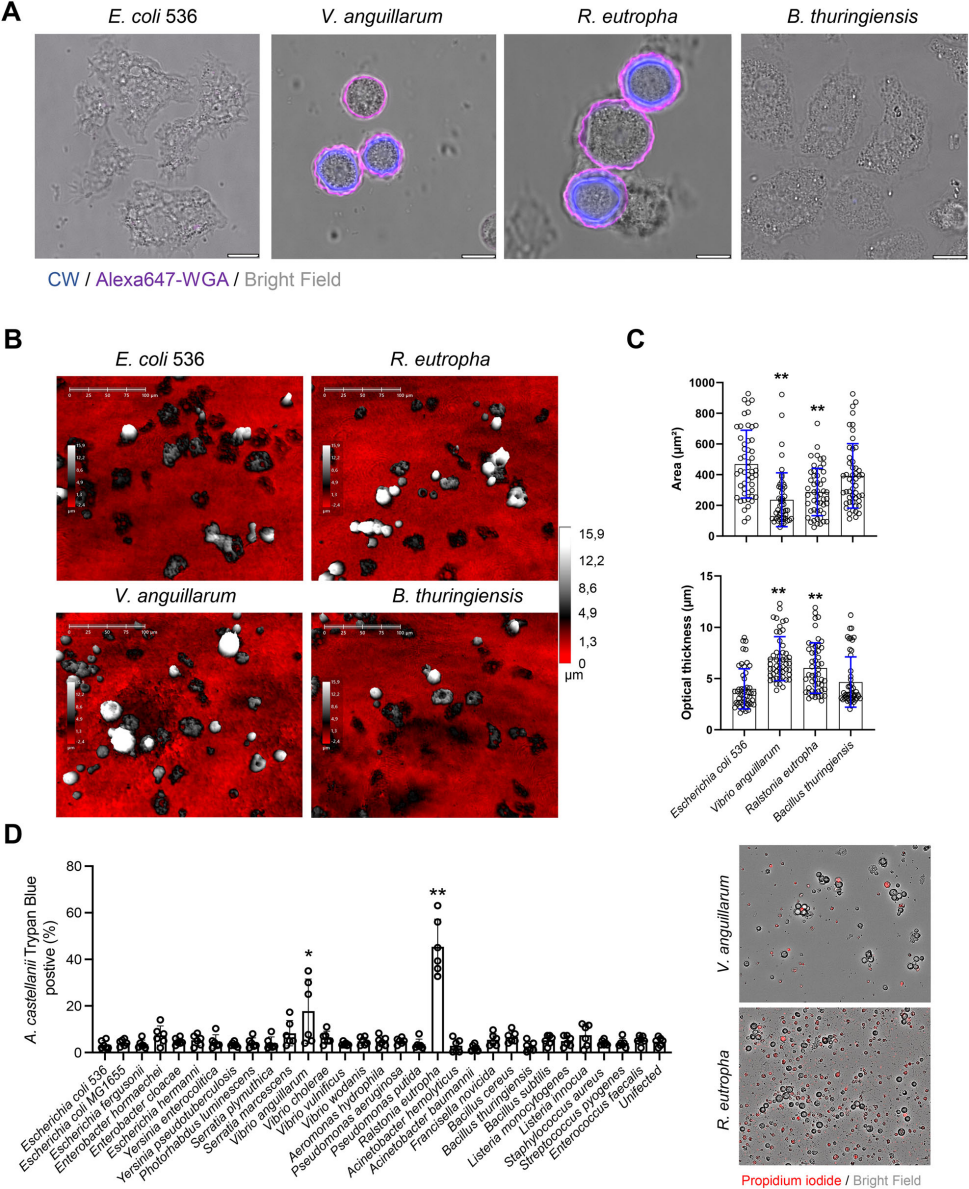
Cyst induction, changes in morphology and killing of *A. castellanii* are independent of pore-forming toxins produced by the bacteria. **A** Confocal microscopy of *A. castellanii* infected with various bacterial species (MOI, 1:200) in NSS media for 48 h. The infected *A. castellanii* were fixed and stained with calcofluor white (CW, blue) and Alexa647-WGA (purple). Scale bars = 10 µm. **B**, **C** *A. castellanii* was infected with various bacterial species (MOI, 1:200) in NSS media for 48 h, following data acquisition with holographic microscopy. Histograms indicate changes in the area and optical thickness of *A. castellanii* exposed to selected bacterial species. Data points in the histogram represent individual *A. castellanii* (*n* = 50) from two biologically independent experiments; bar graphs show mean ± s.d. Significance was determined from replicates using a one-way analysis of variance (ANOVA) with Dunnett’s post-test against *E. coli* 536. **p < 0.01, *p ≤ 0.05. **D** Cell viability was quantified by incubating the cells with Trypan Blue or staining the cells with propidium iodide (images to the right). Histogram indicates the number of Trypan Blue positive *A. castellanii* killed by various bacterial species. Data points represent six replicates from two biologically independent experiments; bar graphs show mean ± s.d. Significance was determined from biological replicates using a non-parametric *t*-test. *p ≤ 0.05, **p ≤ 0.01. Significance was determined from replicates using a one-way analysis of variance (ANOVA) with Dunnett’s post-test against *E. coli* MG1655. **p < 0.01, *p ≤ 0.05.

The ability of selected bacterial species to induce cyst formation in *A. castellanii* was further confirmed by confocal microscopy (Fig. 2A). *A. castellanii* samples infected with various bacterial species, including *E. coli* 536, *V. anguillarum*, *R. eutropha*, and *B. thuringiensis*, were stained with Calcofluor White (CW) and wheat germ agglutinin-647 (WGA-647), which binds to the glycoproteins in the cyst wall [30] (Fig. 2A). Consistent with the HTI screening results, selected bacterial species, *V. anguillarum* and *R. eutropha* induced cyst formation in *A. castellanii*, whereas *E. coli* 536 and *B. thuringiensis* did not. In conclusion, our findings suggest that there is no significant correlation between cyst formation in *A. castellanii* and the presence of bacteria known for producing pore-forming toxins.

### Cyst inducing bacteria decreased the motility of *A. castellanii*

Phase holographic imaging and quantitative phase imaging techniques have been utilized for label-free, extended time-lapse imaging of mammalian cells [31]. In our study, we employed holographic microscopy to explore the morphological changes in *A. castellanii* upon exposure to selected bacterial species (Fig. 2B, C). Our observations revealed that when *A. castellanii* was co-cultured with *E. coli* 536 or *B. thuringiensis*, there were no significant changes in the area and thickness of *A. castellanii*. In contrast, co-culturing with *V. anguillarum* and *R. eutropha* caused a noticeable reduction in the total area (µm²) and an increase in cell thickness (µm) of *A. castellanii* (Fig. 2C). To further explore changes in motility of *A. castellanii* in response to cyst-inducing bacteria (*V. anguillarum* or *R. eutropha*) versus non-cyst-inducing bacteria (*E. coli* 536 or *B. thuringiensis*), we conducted label-free phase holographic imaging coupled with single-cell tracking analysis. Our results indicated an increase in the average motility of *A. castellanii* when infected with *E. coli* 536 or *B. thuringiensis* compared to infections with *V. anguillarum* or *R. eutropha* (Supplementary Movies 1–4 and Supplementary Fig. 2A, B). Collectively, these findings suggest that cyst-inducing bacteria significantly reduce the motility of *A. castellanii*.

### Cell lysis in *A. castellanii* is independent of PFTs produced by the bacteria

To determine whether PFT-producing bacteria induce cell lysis in *A. castellanii*, we infected the amoebae with various bacterial species for 48 h in NSS and assessed membrane integrity using Trypan Blue staining. Consistent with the cyst induction results, *V. anguillarum* and *R. eutropha* were observed to cause the highest degree of cell lysis in infected *A. castellanii* (Fig. 2D). This observation was further validated by staining with propidium iodide, which confirmed the cytotoxic effects of *V. anguillarum* and *R. eutropha* (Fig. 2D, right panel).

**Fig. 3:**
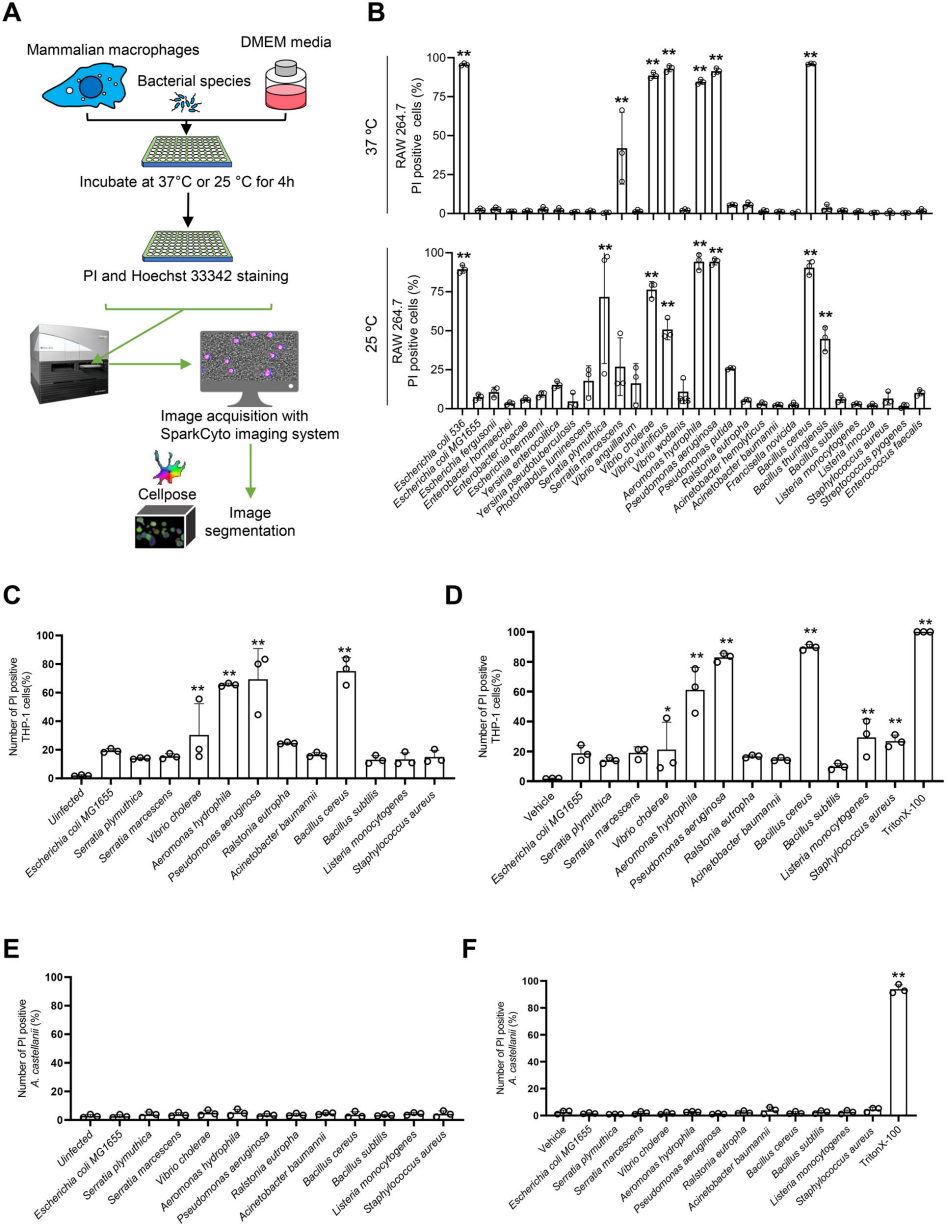
Killing of macrophages is associated with PFT-producing bacteria. **A** Schematic overview of RAW 264.7 murine macrophages infected with various bacterial species (MOI, 1:50) for 4 h in a 96-well plate. **B** RAW 264.7 macrophages were infected with various bacterial species (MOI, 1:50) in complete DMEM media for 4 h at 37 °C and 25 °C, followed by staining with propidium iodide (red). Nuclei were counterstained with Hoechst 33342 (blue). Images were acquired using Spark Cyto imaging system, followed by image segmentation using Cellpose. The number of propidium iodide positive dead cells was normalized against Hoechst 33342-positive total number of cells. Histograms show the percentage of cell death induced by various bacterial species. Data points represent three replicates from independent experiments; bar graphs show mean ± s.d. Significance was determined from replicates using a one-way analysis of variance (ANOVA) with Dunnett’s post-test against *E. coli* MG1655. **p < 0.01, *p ≤ 0.05. **C** THP1 monocytes were differentiated into macrophages with PMA (100 ng/mL, 24 h). The following day, THP1 macrophages were infected with various bacterial species (MOI, 1:50) in complete DMEM media for 4 h at 37 °C, followed by staining with propidium iodide. Nuclei were counterstained with Hoechst 33342. Images were acquired using Spark Cyto imaging system, followed by image segmentation using Cellpose. The number of propidium iodide positive dead cells were normalized against the number of Hoechst 33342-positive cells. Data points represent three replicates from independent experiments; bar graphs show mean ± s.d. Significance was determined from replicates using a one-way analysis of variance (ANOVA) with Dunnett’s post-test against uninfected. **p < 0.01, *p ≤ 0.05. **D** THP-1 macrophages were treated with bacteria cell free supernatant (20%) for 4 h in complete DMEM media at 37 °C. Following treatment cells were stained with propidium iodide and Hoechst 33342. Histograms indicate percentage of cell death induced by the bacterial supernatant. Data points represent three replicates from independent experiments; bar graphs show mean ± s.d. Significance was determined from replicates using a one-way analysis of variance (ANOVA) with Dunnett’s post-test against the vehicle. **p < 0.01, *p ≤ 0.05. **E** *A. castellanii* were infected with various bacterial species (MOI, 1:50) in DMEM media for 4 h at 37 °C, followed by staining with propidium iodide. Histograms indicate percentage of cell death induced by various bacterial species. Data points represent three replicates from independent experiments; bar graphs show mean ± s.d. Significance was determined from replicates using a one-way analysis of variance (ANOVA) with Dunnett’s post-test against uninfected. **p < 0.01, *p ≤ 0.05. **F** *A. castellanii* were treated with bacteria cell free supernatant (20%) for 4 h in complete DMEM media at 37 °C, followed by staining with propidium iodide. Histograms indicate the percentage of cell death induced by the bacterial supernatant. Data points represent three replicates from independent experiments; bar graphs show mean ± s.d. Significance was determined from replicates using a one-way analysis of variance (ANOVA) with Dunnett’s post-test vehicle. **p < 0.01, *p ≤ 0.05.

Given the functional similarities between mammalian macrophages and *A. castellanii* in terms of phagocytic behaviors, including transcriptional, post-transcriptional, and cellular processes [32], we extended our investigation to study the cytotoxic effects of both pathogenic and non-pathogenic bacterial species on mammalian phagocytic cells. For this, we used RAW 264.7 murine macrophages and THP-1 human macrophages (Fig. 3A–D). RAW 264.7 macrophages were exposed to different bacterial species at an MOI of 50 for 4 h at either 37 °C or 25 °C in DMEM media. The cytotoxic response was quantified by staining the cells with propidium iodide to detect dead cells and Hoechst 33342 to quantify the total number of cells. Imaging was conducted using the Spark Cyto system, and images were processed for segmentation using Cellpose 2. The data suggested that most of the tested PFT-producing bacteria (including *E. coli* 536, *Serratia plymuthica*, *S. marcescens*, *V. cholerae*, *Vibrio vulnificus*, *A. hydrophilia*, *P. aeruginosa*, *B. cereus*, and *Bacillus thuringiensis*) induced cell death in murine RAW 264.7 macrophages at both 37 °C and 25 °C (Fig. 3A, B). While most bacterial species exhibited comparable levels of toxicity towards the RAW 264.7 cells at both temperatures, there were notable exceptions. For instance, *S. plymuthica* (71.75% vs. 1%) and *B. thuringiensis* (45% vs. 2.5%) caused higher level of cell death at 25 °C than at 37 °C, while *S. marcescens* (27% vs. 42%) and *V. vulnificus* (51% vs. 93%) caused slightly lower cell death in the cultures of infected cells at 25 °C than at 37 °C (Fig. 3B and Supplementary Fig. 3). Interestingly, *A. castellanii* exposed to PFT-producing bacteria failed to induce cell death under similar experimental conditions (Fig. 3E). To rule out the possibility that PFTs were not expressed by the bacteria during co-incubation with *A. castellanii*, we isolated supernatants from overnight bacterial cultures and exposed both THP1 macrophages and *A. castellanii* to the bacteria-free supernatants. We used TritonX-100 as a positive control for these experiments (Fig. 3D, F). Consistent with the bacterial infection experiments, the supernatants induced cell death in THP-1 macrophages but failed to cause cell death in *A. castellanii* (Fig. 3D, F). Together these findings suggest that, unlike mammalian macrophages, *A. castellanii* resists the bacterial PFT-mediated cell death.

### *B. cereus* delivers the components of the tripartite toxin to mammalian macrophages and *A. castellanii*

**Fig. 4:**
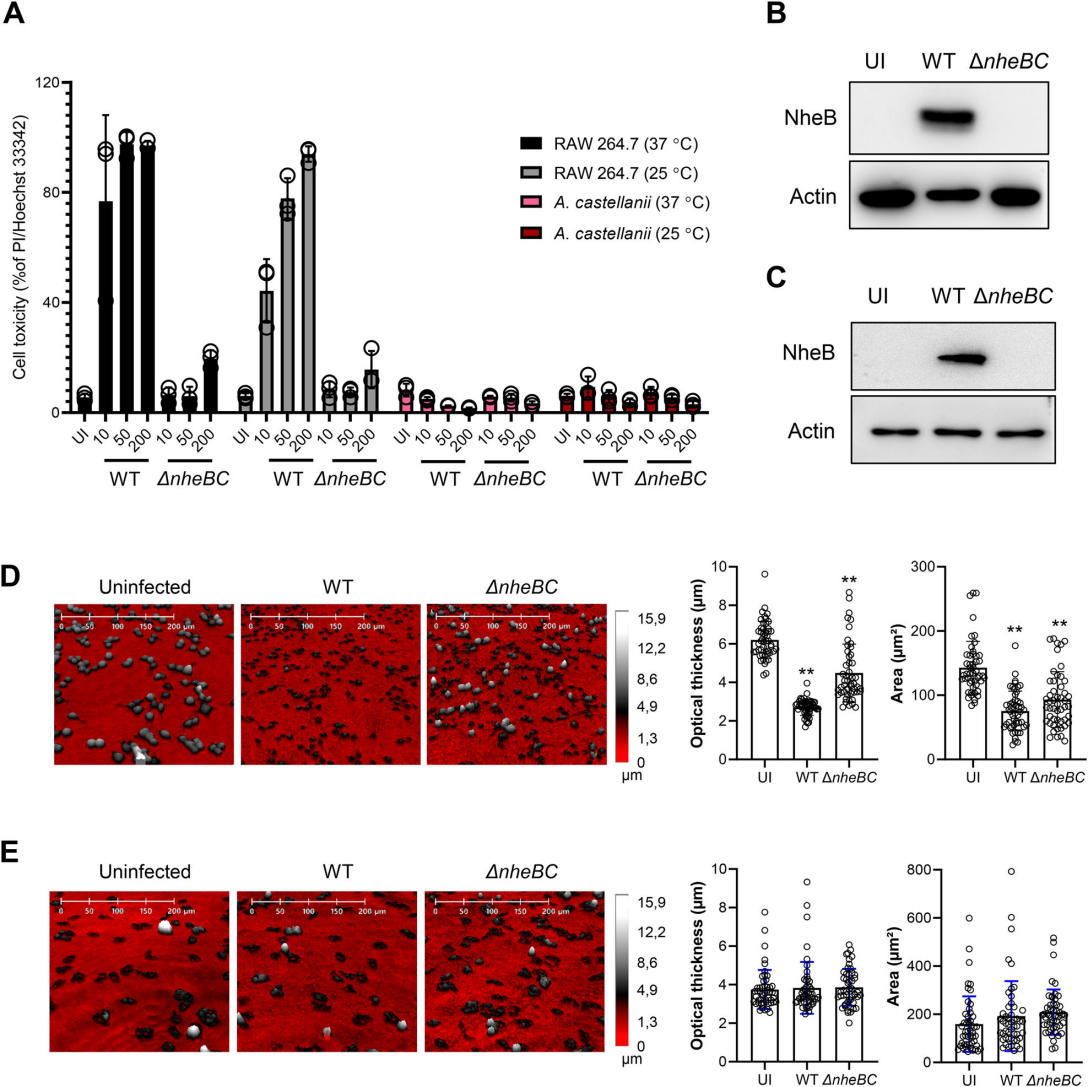
*B. cereus* delivers the components of the NheABC to macrophages and *A. castellanii.* **A** *A. castellanii* and RAW 264.7 macrophages were infected with wild type *B. cereus* and its isogenic mutant *ΔnheBC* strain at an increasing MOI in DMEM media for 4 h at 37 °C and 25 °C, followed by staining with propidium iodide. Histograms show the percentage of cell death induced by wild type *B. cereus* and its isogenic mutant *ΔnheBC* strain. Data points represent three replicates from independent experiments; bar graphs show mean ± s.d. Western blot analysis shows NheB detection in RAW 264.7 macrophages (**B**) and *A. castellanii* (**C**) infected with wild type *B. cereus* and its isogenic mutant Δ*nheBC* (MOI, 1:50) for 4 h in DMEM media at 37 °C. Actin was used as a loading control. Changes in cell morphology of RAW 264.7 macrophages (**D**) and *A. castellanii* (**E**) were recorded by holographic microscopy. Cells were infected with wild type *B. cereus* and its isogenic mutant *ΔnheBC* (MOI, 1:50) in DMEM media for 4 h at 37 °C. Representative holographic microscopy images of RAW 264.7 macrophages and *A. castellanii* after 4 h, infected with bacteria at 37 °C in DMEM media. The color gradients in the images, from red to white, indicate varying levels of cell thickness, with white representing the thickest cells. The histograms to the right in **D** and **E** indicate changes in area and optical thickness. Data points in the histogram represent individual RAW 264.7 macrophages and *A. castellanii* (*n* = 50); bar graphs show mean ± s.d. Significance was determined from replicates using a one-way analysis of variance (ANOVA) with Dunnett’s post-test against uninfected. **p < 0.01, *p ≤ 0.05.

We observed a strong cytotoxic response in both murine and human macrophages when exposed to *B. cereus* (Fig. 3B–D). To address whether *B. cereus* may deliver the components of the NheABC tripartite pore-forming toxin to intoxicated macrophages or *A. castellanii*, we conducted experiments using the *B. cereus* NVH0075/95 strain. Our results demonstrated an MOI-dependent increase in the cell death of RAW 264.7 macrophages at both 37 °C and 25 °C upon infection with the wild type *B. cereus* (Fig. 4A). In contrast, the isogenic mutant Δ*nheBC* strain, which lacks the NheB and NheC components of the tripartite toxin, exhibited minimal cytotoxicity in macrophages (Fig. 4A). Notably, under the similar experimental conditions, both wild type *B. cereus* and its isogenic mutant Δ*nheBC* strain failed to induce cell death in *A. castellanii* (Fig. 4A). To further investigate the delivery of components of the Nhe tripartite toxin, we performed western blot analysis to detect the NheB protein, a key component of the tripartite toxin, in the infected RAW 264.7 macrophages and *A. castellanii* upon infection with the wild type *B. cereus* and its isogenic mutant Δ*nheBC* strain. The results confirmed that NheB was successfully delivered to both RAW 264.7 macrophages and *A. castellanii* (Fig. 4B, C and Supplementary Fig. 4). Consistent with the cell death data, we also observed significant changes in the cell morphology of the RAW 264.7 macrophages infected with both the wild type *B. cereus* and its isogenic mutant Δ*nheBC* strain (Fig. 4D). However, no significant morphological changes were observed in *A. castellanii* when infected with either the wild type *B. cereus* or the isogenic mutant Δ*nheBC* strain (Fig. 4E).

## Discussion

Employing a cutting-edge high-throughput imaging (HTI) screening methodology, our study unravels a complex interplay that challenges the conventional assumption that PFTs contribute to host cell death by lysing membranes in host-pathogen interactions [2]. This research represents a pioneering systematic approach using deep learning tools to examine cyst induction in *A. castellanii* by various bacterial species. Moreover, this study investigates the role of PFTs-producing bacteria in killing of *A. castellanii* and macrophages, revealing previously unrecognized differences in susceptibility between these hosts. Our study presents a significant paradigm shift in understanding the interactions between *A. castellanii* and bacterial PFTs. Even though previous studies have shown the importance of bacterial PFTs in cyst induction and cell death of *A. castellanii* [22, 33], in the present study we demonstrated that bacterial PFTs play a minimal role in such interactions.

Previously, it was suggested that bacteria utilize PFTs to disrupt non-acidic vacuoles within *A. castellanii*, facilitating their escape into the extracellular space and potentially leading to the amoeba’s death [22, 33]. Earlier studies have highlighted the significance of different bacterial toxins in affecting *A. castellanii*, particularly focusing on the impact of *V. cholerae*’s PFT, VCC, the Shiga toxin produced by *E. coli* O157:H7, and various effectors like ExoS, ExoT, and ExoU from *P. aeruginosa* [22, 33–35]. Contrary to the established view, our findings reveal a surprising disconnect between the presence of PFT-producing bacteria and their expected biological effects in *A. castellanii*. Specifically, there was no direct correlation between PFT-producing bacteria and the induction of cell death or cyst formation in *A. castellanii*.

*A. castellanii* serve as valuable model organisms for studying host-pathogen interactions due to their predatory phagocytic nature, resembling that of more complex immune cells like macrophages [36, 37]. A potential difference between the lipid composition of *A. castellanii* and the mammalian cells is the presence of different types of sterols. Previous studies have reported that ergosterol and 7-dehydrostigmasterol are the major sterols of *A. castellanii*, while cholesterol is the principal membrane sterol found in mammalian cells [38]. Cholesterol in the cell membrane plays a crucial role in the oligomerization of various α-PFTs and β-PFTs produced by the pathogenic Gram-negative and Gram-positive bacteria [4, 9, 39–41]. When these toxins bind to cholesterol in the membrane, they undergo conformational changes that allow them to oligomerize, forming a pre-pore complex. This complex eventually inserts into the membrane, creating a pore that can ultimately cause lysis of the mammalian cell membrane [9, 40, 41]. The difference in the sterol composition may therefore possibly explain the resistance of *A. castellanii* to broad range of bacterial PFTs produced by the pathogenic bacteria, highlighting the need for further studies into the sterol-dependent mechanisms of PFT function. Interestingly, despite this resilience, *A. castellanii* remains susceptible to cyst induction by certain bacteria, such as *V. anguillarum* and *R. eutropha*. These bacteria were among the most effective in triggering cyst formation, an adaptive survival mechanism, while other environmental bacteria like *S. marcescens*, *B. cereus*, and *P. aeruginosa* induced significantly lower cyst formation. This differential response suggests that the ability to induce cysts is not solely dependent on PFT production but may involve other virulence strategies or secreted factors unique to these bacteria. Another aspect worth noting is the ecological significance of these interactions. The ability of *A. castellanii* to resist PFT-induced cell death while forming cysts could represent an evolutionary adaptation that helps the amoeba survive in harsh environments populated by diverse bacterial communities. This trait may allow *A. castellanii* to act as a reservoir for certain bacterial pathogens, potentially aiding in their persistence and dissemination in the environment.

Among the various bacterial species used in the current study, *B. cereus* Frankland and Frankland 1887 strain induced the maximum cell death in the infected macrophages. It produces two tripartite toxins (NheABC and Hbl-BL_1_L_2_) and a single component toxin, CytK for efficient killing of mammalian cells [42–44]. Additionally, it has been suggested that the sphingomyelinase (SMase) produced by *B. cereus* has the ability to kill mammalian cells [43]. For understanding the role of NheABC PFTs in killing of macrophages and *A. castellanii* we used *B. cereus* NVH 0075/95 strain, which expresses the NheABC tripartite toxin and sphingomyelinase (SMase) as major virulence factors against target mammalian cells [45–47]. The minimal cytotoxic response observed in the macrophages infected with the otherwise isogenic mutant Δ*nheBC* lacking the NheB and NheC components of the NheABC tripartite toxin suggests that in addition to NheABC, SMase also contributes to cell death in response to *B. cereus* infection [43]. While most of the PFT-producing bacteria caused significant cytotoxicity in macrophages, certain species like *Y. enterocolitica* and *Y. pseudotuberculosis* did not induce cell death in RAW 264.7 macrophages, likely due to inactive type 3 secretion systems (T3SS). This finding aligns with previous reports that T3SS activation requires specific environmental cues, such as calcium or serum components, to deliver cytotoxic effector molecules to the infected cells [48].

Our study also underscores the value of unbiased, image-based approaches combined with advanced deep learning tools like Cellpose and StarDist. These tools enabled accurate segmentation and analysis of *A. castellanii* trophozoites and cysts, overcoming the limitations of traditional methods in handling complex morphological features and clustered cells. Although Cellpose and StarDist segmentation of the *A. castellanii* trophozoites and cysts is highly accurate, densely packed or partly overlapping cells are sometimes difficult to segment with currently available Cellpose and StarDist models. Cell segmentation is a critical step in many downstream applications of microscopy-based HTI screening across biological research. Deep learning tools like Cellpose and StarDist have revolutionized this process by providing unprecedented accuracy and efficiency, making them indispensable for analyzing data generated from HTI screening. To the best of our knowledge, this is the first study to apply these advanced deep learning tools to investigate the interaction between bacterial species and *A. castellanii*. The application of these tools for detecting specific features of *A. castellanii* during bacterial interactions represents a significant advancement, opening new opportunities for research in environmental science, microbial ecology, and public health.

Our experiments demonstrated that *V. anguillarum*, an aquatic pathogen, and *R. eutropha*, a widely distributed environmental bacterium, induced the highest levels of cyst formation in *A. castellanii*. Our findings suggest that *V. anguillarum* and *R. eutropha* may have evolved to effectively encounter the predation of *A. castellanii* by inducing cysts formation or killing it. Further investigation into the interactions between *A. castellanii* and *V. anguillarum* could provide deeper insights into the virulence mechanisms of *V. anguillarum*, a pathogen with significant economic impact in the aquaculture industry [49].

In conclusion, our findings challenge the conventional understanding of bacterial PFTs in interactions with *A. castellanii*. The amoeba exhibited remarkable resilience to PFTs, which typically are cytolytic and harmful to mammalian cells. This raises intriguing questions about whether *A. castellanii* has evolved specific defense mechanisms that play a more intricate ecological role, potentially shaping its interactions with bacteria and contributing to the maintenance and dissemination of pathogens in the environment. Our results underscore the importance of exploring protozoan defense mechanisms and the broader context of microbial ecology, providing a foundation for further research into these complex relationships.

## Materials and methods

### Growth conditions for bacterial strains

All bacterial strains used in this study are listed in Table 1. The bacterial strains were grown on Luria/Lysogeny agar (LA) plates and incubated overnight under specific temperature conditions. Bacterial strains were incubated at 37 °C, with exceptions for *Vibrio anguillarum*, which was incubated at 30 °C and *Vibrio wodanis,* which was incubated at 25 °C.

**Table 1:** Bacterial species used in the study.

Strains	Description/Relevant characteristics	Reference
*Escherichia coli* 536	UPEC strain (O6:K15:H31) wild type	[29]
*Escherichia coli* MG1655	*E. coli* K-12 wild type	[52]
*Escherichia coli* MC1061	*E. coli* K-12 *araD139 Δ(ara leu)7697 ΔlacX74 galU galK hsr hsm*^*+*^ *strA*	[53]
*Escherichia fergusonii*	Wild type	DSM 13698
*Enterobacter hormaechei*	Wild type	DSM12409
*Enterobacter cloacae*	Wild type	CCUG 59573
*Escherichia hermannii*	Wild type	DSM 4560
*Yersinia enterocolitica*	Wild type	W22703
*Yersinia pseudotuberculosis*	*yadA*::Tn*5*, Km^R^ YPIII/pIB102	[54]
*Photorhabdus luminescens*	Wild type	TT01
*Serratia plymuthica*	Wild type	CCUG 61396
*Serratia marcescens*	Wild type	ATCC 274
*Vibrio anguillarum* NB10	Isolated from the Gulf of Bothnia	[55]
*Vibrio cholerae* A1552	Serogroup O1, Rif^R^	[56]
*Vibrio vulnificus* E-86	Isolated from diseased European Eel, Spain	[57]
*Vibrio wodanis*	Origin 88/411 Norway	This study
*Aeromonas hydrophila* AH3	Serotype O:34	[58]
*Pseudomonas aeruginosa* PAO1	Clinical isolate from wound infection	[59]
*Pseudomonas putida* KT2440	Wild type	ATCC 47054
*Ralstonia eutropha* H16	Wild type	DSM 428
*Acinetobacter haemolyticus*	Wild type	DSM 6962
*Acinetobacter baumannii* 17978	Wild type	ATCC17978
*Bacillus cereus*	Wild type (Frankland and Frankland 1887)	DSM31
*Bacillus cereus*	Wild type (NVH0075/95)	[47]
*Bacillus cereus ΔnheBC* mutant	*ΔnheBC* (NVH0075/95)	[60]
*Bacillus thuringiensis* USDA H522	Wild type	ATCC 35646
*Bacillus subtilis* 168	BGSC, Bacillus Genetic Stock Centre, Columbus	[61]
*Streptococcus pyogenes* AP1	Clinical isolate: Serotype M1	[62]
*Enterococcus faecalis* OG1RF	Parent strain, Rif^R^, Fus^R^	[63]
*Staphylococcus aureus*	Clinical isolate from wound infection	ATCC 29213
*Listeria monocytogenes* EGDe	Wild type	K. Boor
*Listeria innocua* BUG499	Wild type	[64]

### Growth conditions for *A. castellanii* and RAW 264.7 and THP-1 cell cultures

*Acanthamoeba castellanii* (ATCC strain 30010) was grown aerobically at 30 °C in tissue culture flasks with ventilated caps (Sarstedt) using peptone yeast glucose (PYG) medium (ATCC medium 712) as substrate. The PYG medium was prepared by dissolving 20 g l^−1^ protease peptone extract, 1 g l^−1^ yeast extract, 0.9 g l^−1^ glucose, and inorganic salts [0.44 mM CaCl_2_•2H_2_O, 4.44 mM MgSO_4_·7H_2_O, 2.78 mM Na_2_HPO_4_·7H_2_O,2.78 mM KH_2_PO_4_, 5.28 mM HOC(COONa)(CH_2_COONa)_2_·2H_2_O, 0.006 mM Fe(NH_4_)_2_(SO_4_).6H_2_O].

To compare the effects of PFTs on *A. castellanii* with effects on mammalian macrophages, we used the murine RAW 264.7 macrophages (ATCC® TIB-71 ™) and human THP-1 monocytes (ATCC® TIB-202™) cell lines that were maintained in Dulbecco’s modified Eagle medium (DMEM) (Sigma-Aldrich) supplemented with 10% fetal bovine serum (FBS), 1% penicillin/streptomycin, and non-essential amino acids at 37 °C with 5% CO_2_.

### Bacterial infections and cell toxicity

The bacterial strains were grown on Luria Agar (LA) plates overnight at their respective optimal incubation temperatures, as specified above. *A. castellanii* (5 × 10^3^) were seeded in a 96-well plate in PYG medium and incubated at 25 °C overnight without shaking. The next day, *A. castellanii* was infected separately with each bacterial species at a multiplicity of infection (MOI) of 1:200 in a nine-salt solution (NSS) for 48 h at 25 °C. NSS was prepared by dissolving 17.6 g NaCl, 1.47 g Na2SO4, 0.08 g NaHCO_3_, 0.25 g KCl, 0.04 g KBr, 1.87 g MgCl_2_•6H_2_O, 0.45 g CaCl_2_•2H_2_O, 0.01 g SrCl_2_•6H_2_O, and 0.01 g H_3_BO_3_ in one liter of distilled water [50].

At the end of the bacterial infection, cell toxicity in *A. castellanii* was assessed by incubating the amoeba with propidium iodide (PI, 3 μg/mL) for 1 h, followed by imaging using the Spark Cyto imaging system (Tecan). Cell toxicity was further evaluated by manually counting trypan blue positive cells.

The murine RAW 264.7 macrophages were grown in DMEM medium supplemented with 10% fetal bovine serum (FBS) and incubated at 37 °C overnight in an incubator with continuous flow of 5% CO_2_. The following day, cells were infected with bacteria (MOI, 1:50) for 4 h. Cell toxicity in RAW 264.7 macrophages was measured by staining the cells with a mixture of PI (1 µg/mL) and Hoechst 33342 (2 µM) for 30 min, followed by image acquisition using the Spark Cyto imaging system (Tecan).

THP-1 monocytes (1 × 10^4^) were grown in DMEM medium supplemented with 10% FBS and treated with phorbol 12-myristate 13-acetate (PMA) at a concentration of 100 ng/ml to differentiate them into macrophages for 48 h at 37 °C. Differentiated THP-1 macrophages were then infected with bacteria (MOI, 1:50) for 4 h, following cell toxicity measurement.

To investigate the effects of bacteria-free supernatants on *A. castellanii* and THP-1 macrophages, selected bacterial species were cultured in LB overnight. The cultures were centrifuged at 5000 rpm for 10 min, and the supernatant was filtered through a 0.22 µm polyether sulfone (PES) syringe filter. THP1 macrophages and *A. castellanii* were treated with 20% bacteria-free supernatants in complete DMEM media for 4 h. LB media (20%) was used as a vehicle control. Cell toxicity in THP-1 cells was measured by staining with a mixture of PI (1 µg/mL) and Hoechst 33342 (2 µM) for 30 min, followed by image acquisition using the Spark Cyto imaging system (Tecan).

For the infection experiments with wild type *B. cereus* and its isogenic mutant Δ*nheBC* strain, *A. castellanii* and RAW 264.7 macrophages were infected at increasing MOIs in DMEM media for 4 h at 37 °C and 25 °C, followed by staining with propidium iodide for 30 min at the respective temperatures. Images were acquired with Spark Cyto imaging system (Tecan).

### Deep-learning tools used for image analysis

Images acquired with the Spark Cyto imaging system were analyzed using either the “Analyze Particles” plugin available in Fiji [26] or two deep-learning tools: StarDist [28] and Cellpose [25]. The “Analyze Particles” function in Fiji was employed to segment and quantify the *A. castellanii*, specifying that only particles larger than 40 pixels^2^ (6.32 μm^2^) were counted. The output of this function was a comma-separated values (CSV) file containing the names of each image and the corresponding number of particles detected.

The deep-learning tool StarDist, integrated as a Fiji plugin, specializes in segmenting round objects such as cell nuclei and other subcellular structures. The plugin was run with the default settings using the “Versatile” model [28]. Images were preprocessed by converting them to 8-bit format in Fiji before applying the StarDist plugin. To facilitate batch processing of large datasets, a custom macro code was developed. This macro code applied the StarDist plugin to each image in a designated folder, saved the segmentation results, and compiled the outputs into a single CSV file for subsequent analysis. Similar to “Analyze Particles”, only segmented particles larger than 40 pixels^2^ (6.32 μm^2^) were included in the analysis. Cellpose, a recently developed deep-learning tool capable of segmenting a variety of objects within an image was employed. A customized python code was developed for batch processing of images stained with calcofluor-white-, acridine orange-, and propidium iodide-stained cells. The Cyto2 segmentation model [25] was used for these analyses to ensure accurate and efficient segmentation of the cell populations.

### Confocal microscopy

For fixed cell immunofluorescence, *A. castellanii* (1 × 10^4^/well) was grown overnight in coverslip-bottom 18-well chamber slides (μ-Slide, ibidi) in PYG medium. The following day, *A. castellanii* were washed with NSS and infected with different bacterial species at a MOI of 200 for 48 h at 25 °C and then fixed in 4% paraformaldehyde for 30 min at room temperature, followed by washing with phosphate-buffered saline (PBS; 137 mM NaCl, 2.7 mM KCl, 10 mM Na_2_HPO_4_, 1.8 mM KH_2_PO_4_) for 3 min. For cyst staining, amoeba were stained with calcofluor white (CW; 0.1%) that binds to cellulose in the cyst wall [22], and/or Alexa-647 labeled wheat germ agglutinin (WGA-647; 20 μg/mL), that binds to glycoproteins present in the cyst wall [30] in a blocking solution (5% FBS) for 60 min at room temperature, following washing with PBS (3 min). Images were acquired using a Leica SP8 confocal system (Leica Microsystems) equipped with a HC PL APO 63x/1.40 oil immersion lens. The acquired images were subsequently processed using the LasX software (Leica Microsystems).

### Holographic microscopy

Holographic microscopy was performed using the HoloMonitor® M4 (Phase Holographic Imaging AB) equipped with a motorized stage to investigate the time-dependent changes in *A. castellanii* morphology and motility. *A. castellanii* (5 × 10^5^/well) were grown overnight in 24-well plates using PYG or DMEM medium. The following day, *A. castellanii* were washed with NSS and infected with different bacterial species in NSS media (MOI:200) for 48 h at 25 °C, after which images were acquired using the HoloMonitor® M4. This system generates label-free images reconstructed into three-dimensional holograms. Quantitative measurements, such as average cell thickness and area, were extracted using Hstudio™ software [31], and analyzed using GraphPad Prism software. For the *B. cereus* experiment, RAW 264.7 macrophages and. *A. castellanii* (1 × 10^6^/well) were cultured overnight in DMEM medium in 24-well plates. The following day, *A. castellanii* were infected with *B. cereus* wild type and its isogenic *ΔnheBC* mutant strain at an increasing MOI for 4 h at 37 °C. Images were acquired and processed using similar methods as discussed above.

For the cell tracking experiment, *A. castellanii* was grown in a 24-well plate was washed with NSS media, and infected with the selected bacterial species in NSS media (MOI, 1:200) for 45 h at 25 °C. Image acquisition was performed every 2 min for 3 h using the HoloMonitor M4. This system uses a low power laser (635 nm wavelength, 0.2 mW cm^2^), which minimizes phototoxicity, making it suitable for label-free imaging *A. castellanii* for an extended time. At the end of the experiment, the time-lapse images were analyzed for single-cell tracking using Hstudio™ [31]. Briefly, using the Track Cells module in HStudio, the captured images were segmented, and individual *A. castellanii* were identified and tracked in each frame. A defined threshold in each image was used to select the individual *A. castellanii*. The tracking is semi-automated, using a frame-by-frame algorithm that attempts to find each tracked cell in the next frame based on the centroid position. The user reviews each captured image of the time-lapse and corrects potential tracking errors by identifying and labeling the same cell in all frames. A total of 60 frames were used for cell tracking in the current study. The motility of individual *A. castellanii* at each time point was calculated as the displacement of the object centre between two consecutive images. *A. castellanii* motility at each time point was used to calculate either speed (displacement over time) or total motility (the sum of all motilities over the duration of the imaging).

### Western blot analysis

*Acanthamoeba castellanii* and RAW 264.7 macrophages *(*1 × 10^6^/well) were grown overnight in 6-well plates in DMEM medium. The following day, the cells were infected with the wild type *B. cereus* and its isogenic mutant, *ΔnheBC* (MOI, 1:50) at 37 °C. The infected *A. castellanii* and RAW 264.7 macrophages were washed extensively with PBS (3 times) and lysed in ice-cold lysis buffer containing 20 mM Tris-HCl pH 8, 300 mM KCl, 10% Glycerol, 0.25% Nonidet P-40, 0.5 mM EDTA, 0.5 mM EGTA, 1 mM PMSF, 1X complete protease inhibitor (Roche) and phosSTOP (Roche). The cell lysates were then mixed with 4x sample buffer, boiled for 10 min, separated by SDS-PAGE, and transferred to a nitrocellulose membrane. The membranes were blocked in PBST (PBS supplemented with 0.1% Tween 20) containing 5% skim milk at room temperature (RT) for 1 h. Subsequently, the membranes were incubated overnight at 4 °C with the primary antibodies, anti-NheB (0.2 μg/mL) [51] or anti-actin (Sigma-Aldrich, #A2228, 0.2 μg/mL) in PBST. After washing with PBST, the membranes were incubated with appropriate HRP-conjugated secondary antibodies (Agrisera, #AS09602, 0.2 μg/mL) in blocking buffer (5% skim milk, RT, 1 h). Protein detection was performed using a chemiluminescence reagent (Bio-Rad), and images were acquired using the ImageQuant LAS 4000 instrument.

### Statistical analysis

Data are shown as mean ± s.d. Statistical significance was determined by one-way ANOVA, as indicated in the corresponding Fig. legend. Analysis was performed using GraphPad Prism software. Statistical significance is indicated as *p* ≤ 0.01 (**) or *p* ≤ 0.05 (*).

### Methods

All methods were performed in accordance with the relevant guidelines and regulations.

## Supplementary information

Supplementary Movie 1: Motility of A. castellanii infected with E. coli 536.

Supplementary Movie 2: Motility of A. castellanii infected with R. eutropha.

Supplementary Movie 3: Motility of A. castellanii infected with V. anguillarum.

Supplementary Movie 4: Motility of A. castellanii infected with B. thuringiensis.

Supplementary Information

Uncropped Western Blots

## Data Availability

All data supporting the findings of this study are included in the article and its Supplementary files. Additional data are available from the corresponding author upon request.
