# Supplementary material for: A new understanding of Acanthamoeba castellanii: dispelling the role of bacterial pore-forming toxins in cyst formation and amoebicidal actions
Source: Cell Death Discov. 2025 Feb 19;11:66. doi: 10.1038/s41420-025-02345-8 (PMC11839945; doi:10.1038/s41420-025-02345-8)
Supplement: Supplementary file 5 — Supplementary Information [file 41420_2025_2345_MOESM5_ESM.pdf]

Supplementary Information

A new understanding of *Acanthamoeba castellanii*: Dispelling the role of bacterial pore-forming toxins in cyst formation and amoebicidal actions

Abdelbasset Yabrag[1], Naeem Ullah[1], Palwasha Baryalai[1,2], Irfan Ahmad[1,3], Nikola Zlatkov[1,4], Eric Toh[1,2], Toril Lindbäck[5], Bernt Eric Uhlin[1], Sun Nyunt Wai[1,2], *Aftab Nadeem[1]

[1]Department of Molecular Biology and Umeå Centre for Microbial Research (UCMR), Umeå University, SE-90187 Umeå, Sweden

[2]The Laboratory for Molecular Infection Medicine Sweden (MIMS), Umeå University, SE-90187 Umeå, Sweden

[3]Department of Cell and Molecular Biology, Science for Life Laboratory, Uppsala University, SE-75123 Uppsala, Sweden

[4]Department of Pathology, Medical University of Vienna, Austria

[5]Department of Paraclinical Sciences, Faculty of Veterinary Medicine, Norwegian University of Life Sciences, Ås, Norway

*Corresponding author: Aftab Nadeem, e-mail: aftab.nadeem@umu.se

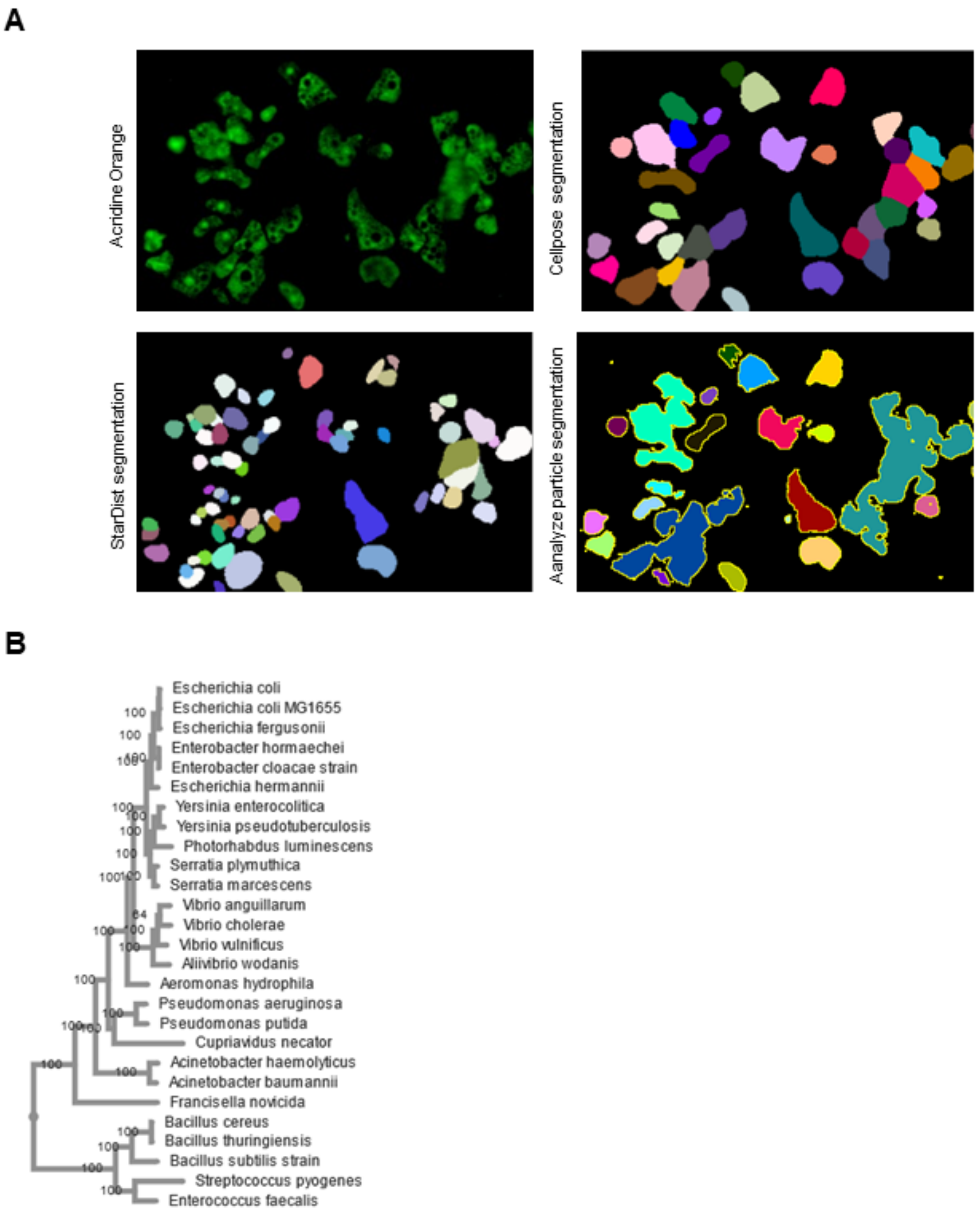

Supplementary Fig. 1: Cellpose outperforms StarDist and Fiji ImageJ in cells segmenting.

A Selected images of *A. castellanii* cells infected with bacteria, processed using the segmentation tools Cellpose 2, StarDist, and the Analyze Particles tool in Fiji ImageJ. **B** Phylogenetic analysis of bacterial species used in the current study.

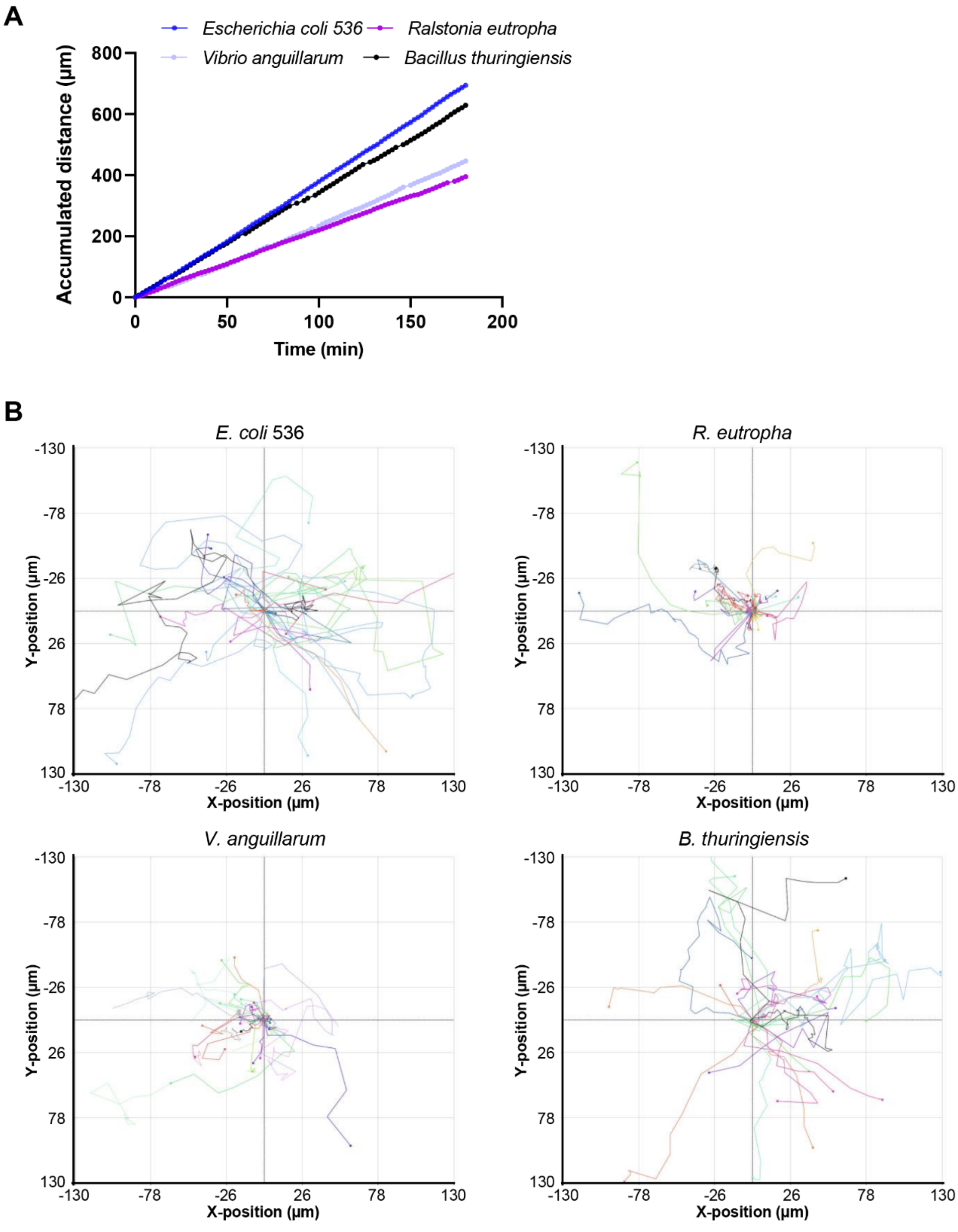

Supplementary Fig. 2: Cysts-inducing bacteria cause a decrease in the motility of *A. castellanii*.

A An accumulated distance covered by the *A. castellanii* exposed to various bacterial species for 45 h, following live cell imaging for 3 h on HoloMonitor M4. Data presented in the plot is a mean of two independent replicates. **B** The directionality of motility of *A. castellanii* is plotted from data presented in (A). Each line graph indicates the motility of a single *A. castellanii* (n = 20).

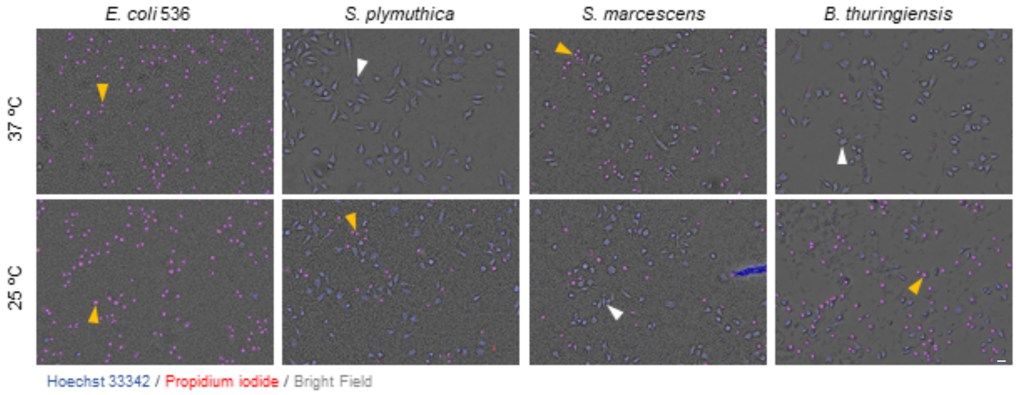

Supplementary Fig. 3: Killing of murine macrophages is associated with PFT-producing bacteria.

Selected images from the screen of RAW 264.7 cells infected with bacteria at 37°C or 25°C. The yellow arrowhead indicates dead cells, while the white arrowhead indicates living cells. Scale bars = 10μm.

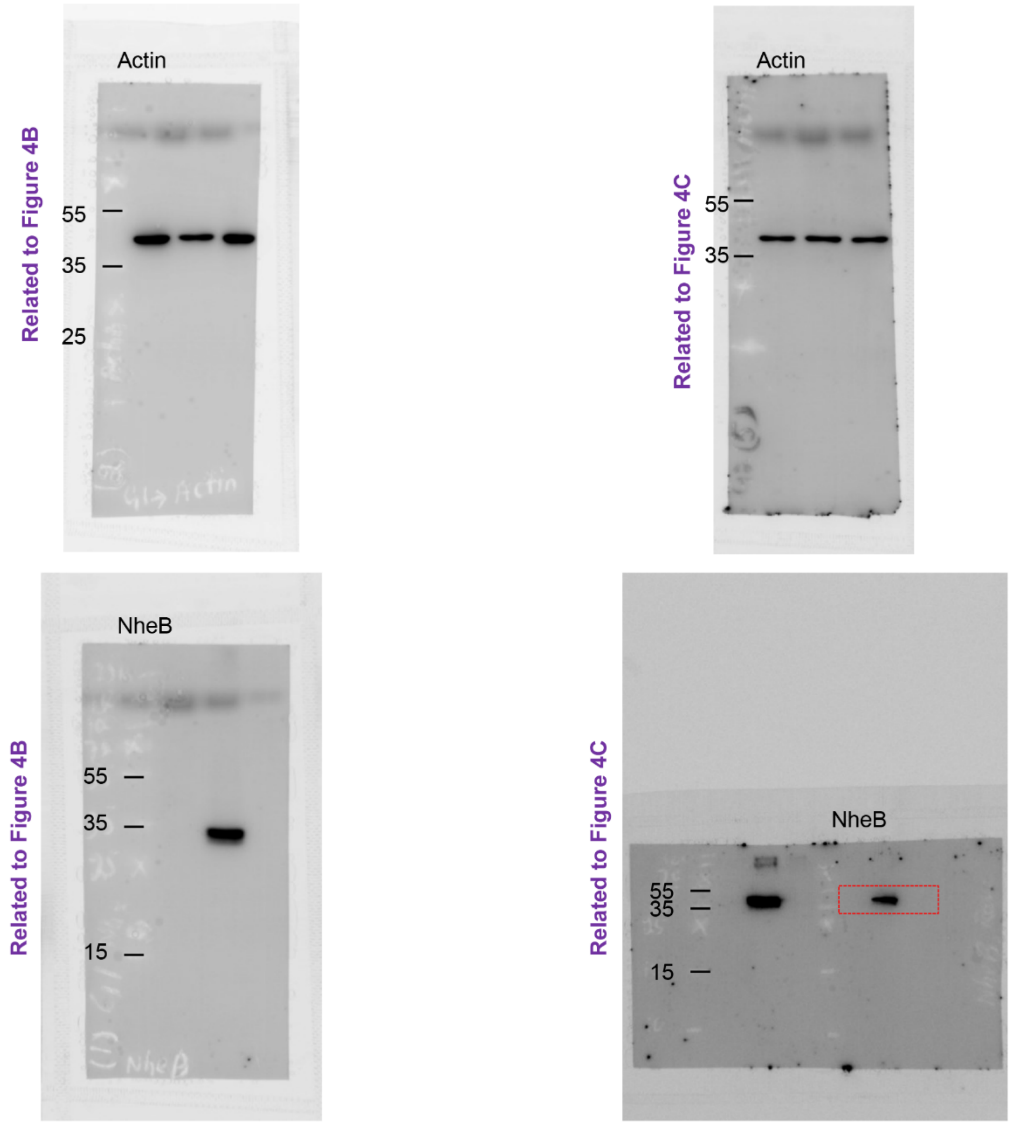

Supplementary Fig. 4: Uncropped western blots
Uncropped western blots presented in Fig. 4. The numbers to the left represent molecular weight markers in kilodalton (kDa). The red box in the last panel to the right indicates the western blot used in Fig. 4C.

Videos Caption

Supplementary Movie 1: Motility of *A. castellanii* infected with *E. coli* 536.
Movie assembled from time-lapse holographic microscopy images (frame rate, 4 fps; total duration, 23 s). The images were obtained from a population of *Acanthamoeba castellanii* that had been infected with *E. coli* 536 for 48 hours (MOI:200). Image acquisition was performed on a HoloMonitor M4, with images taken every 120 s over a period of 180 minutes at room temperature.

Supplementary Movie 2: Motility of *A. castellanii* infected with *R. eutropha.*
Movie assembled from time-lapse holographic microscopy images (frame rate, 4 fps; total duration, 23 s). The images were obtained from a population of *Acanthamoeba castellanii* that had been infected with *R. eutropha* for 48 hours (MOI:200). Image acquisition was performed on a HoloMonitor M4, with images taken every 120 s over a period of 180 minutes at room temperature.

Supplementary Movie 3: Motility of *A. castellanii* infected with *V. anguillarum.*
Movie assembled from time-lapse holographic microscopy images (frame rate, 4 fps; total duration, 23 s). The images were obtained from a population of *Acanthamoeba castellanii* that had been infected with *V. anguillarum* for 48 hours (MOI:200). Image acquisition was performed on a HoloMonitor M4, with images taken every 120 s over a period of 180 minutes at room temperature.

Supplementary Movie 4: Motility of *A. castellanii* infected with *B. thuringiensis.*
Movie assembled from time-lapse holographic microscopy images (frame rate, 4 fps; total duration, 23 s). The images were obtained from a population of *Acanthamoeba castellanii* that had been infected with *B. thuringiensis* for 48 hours (MOI:200). Image acquisition was performed on a HoloMonitor M4, with images taken every 120 s over a period of 180 minutes at room temperature.
