# Supplementary material for: A new understanding of Acanthamoeba castellanii: dispelling the role of bacterial pore-forming toxins in cyst formation and amoebicidal actions
Source: Cell Death Discov. 2025 Feb 19;11:66. doi: 10.1038/s41420-025-02345-8 (PMC11839945; doi:10.1038/s41420-025-02345-8)
Supplement: Supplementary file 6 — Uncropped Western Blots [file 41420_2025_2345_MOESM6_ESM.pdf]

Begränsad delning

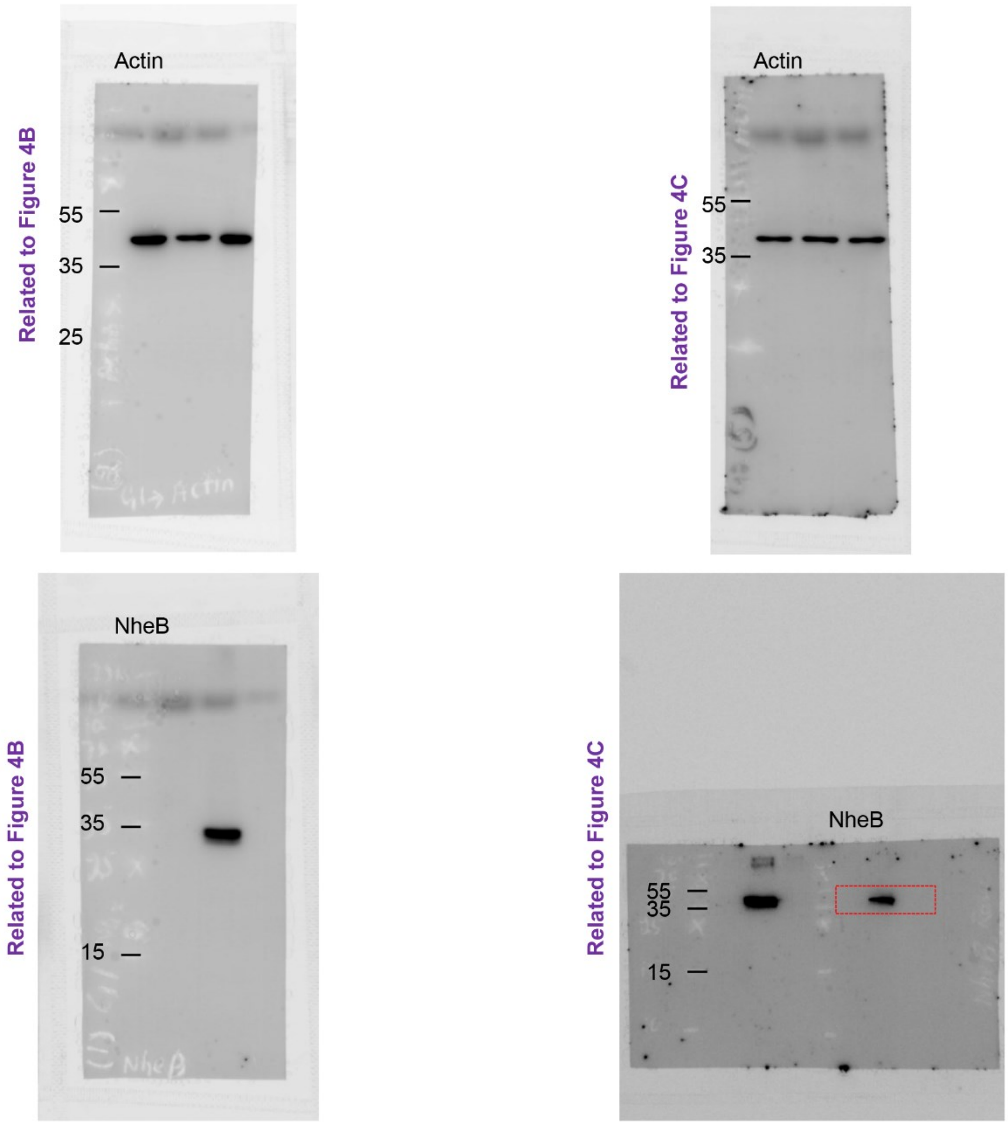

Supplementary Fig. 4: Uncropped western blots

Uncropped western blots presented in Fig. 4. The numbers to the left represent molecular weight markers in kilodalton (kDa). The red box in the last panel to the right indicates the western blot used in Fig. 4C.
